# Sensorineural hearing loss and cognitive impairment: three hypotheses

**DOI:** 10.3389/fnagi.2024.1368232

**Published:** 2024-02-28

**Authors:** He Zhao, Yan Wang, Limei Cui, Hanjing Wang, Sha Liu, Tiantian Liang, Dawei Liu, Jingjing Qiu, Liang Chen, Yan Sun

**Affiliations:** ^1^The Second Medical College, Binzhou Medical University, Yantai, Shandong, China; ^2^Department of Otolaryngology and Head and Neck Surgery, Yantai Yuhuangding Hospital, Qingdao University, Yantai, Shandong, China; ^3^Shandong Provincial Clinical Research Center for Otorhinolaryngologic Diseases, Yantai, Shandong, China; ^4^School of Clinical Medicine, Shandong Second Medical University, Weifang, China

**Keywords:** sensorineural hearing loss, cognitive impairment, cognitive load hypothesis, co-morbidity hypothesis, sensory deprivation hypothesis

## Abstract

Sensorineural hearing loss (SNHL) is a category of hearing loss that often leads to difficulty in understanding speech and other sounds. Auditory system dysfunction, including deafness and auditory trauma, results in cognitive deficits via neuroplasticity. Cognitive impairment (CI) refers to an abnormality in the brain’s higher intellectual processes related to learning, memory, thinking and judgment that can lead to severe learning and memory deficits. Studies have established a strong correlation between SNHL and CI, but it remains unclear how SNHL contributes to CI. The purpose of this article is to describe three hypotheses regarding this relationship, the mainstream cognitive load hypothesis, the co-morbidity hypothesis, and the sensory deprivation hypothesis, as well as the latest research progress related to each hypothesis.

## Introduction

Hearing loss (HL) is a serious condition that not only diminishes a patient’s quality of life but can also lead to lifelong disability. According to a global survey conducted in 2016, HL ranks as the fourth most prevalent disorder and one of the top five contributors to disability (Vos et al., 2017). Sensorineural hearing loss (SNHL), a type of HL, accounts for 90% of reported cases of HL (Li et al., 2017) and leads to difficulties in directly understanding speech and other sounds. Cross-sectional and longitudinal evidence suggests that dysfunction of the auditory system, including deafness and auditory trauma, leads to cognitive deficits that develop via neuroplasticity (Rutherford et al., 2018; Swords et al., 2018). SNHL is generally categorized as age-related hearing loss (ARHL), noise-related hearing loss, or drug-related hearing loss (DRHL).

Cognitive function (*CF*) refers to the processes by which the human brain receives external information, processes it, and converts it into internal mental activity in order to acquire knowledge or apply it. *CF* encompasses memory, language skills, visuospatial abilities, executive functions, computational skills, and comprehension judgments (Liu et al., 2024; Mellow et al., 2024). Cognitive impairment (CI) refers to a pathological process in which abnormalities exist in the higher intellectual processes of the brain related to learning and memory as well as thinking and judgment (Sanford, 2017). Such impairment results in severe deficits in learning and memory accompanied by changes such as aphasia or dysfunction/loss of recognition or behaviors. The pathological changes in the brain associated with CI development primarily involve lesions within the hippocampus.

**Figure 1:**
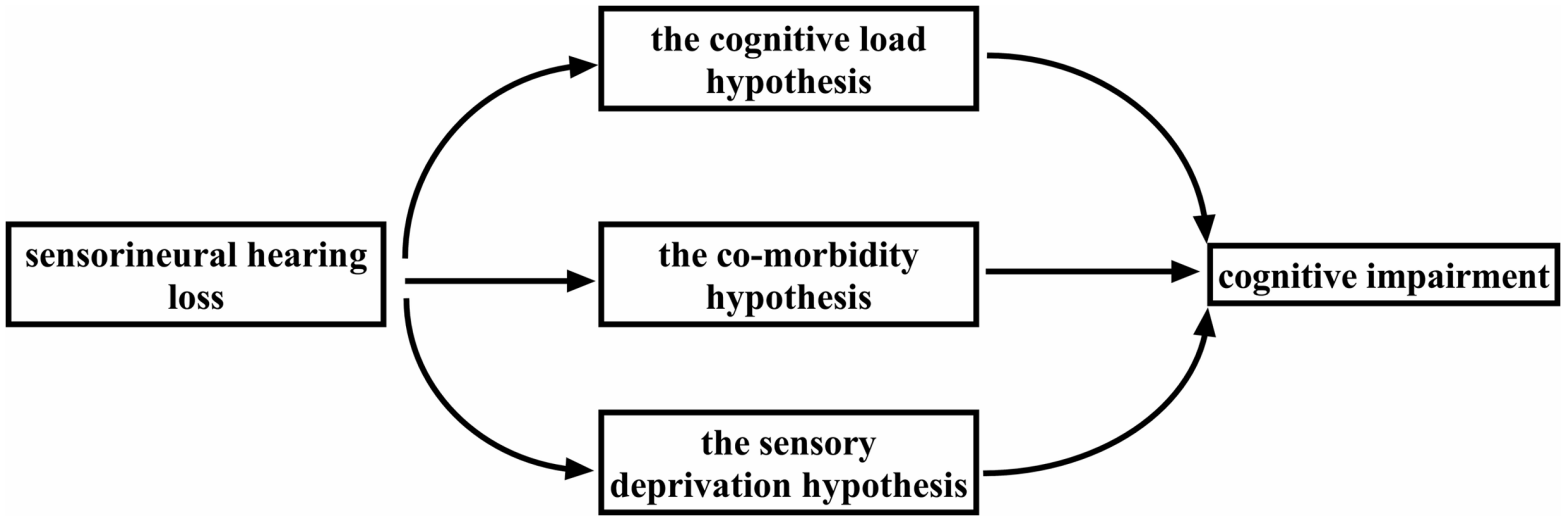
Three hypotheses regarding how SNHL leads to CI: the cognitive load 72 hypothesis, the co-morbidity hypothesis, and the sensory deprivation hypothesis.

The aim of this review is to describe the current hypotheses on how CI results from SNHL and to provide an update on research progress in this field. In some experiments involving mice subjected to inner ear hair cell ablation (Liu et al., 2020; Qian and Ricci, 2020) and in animals chronically exposed to noise (Patel et al., 2022), CI is observed after a period of hearing impairment. RNA sequencing analysis has revealed that ARHL shares a common causative gene with Alzheimer’s disease (AD; Xue et al., 2021). Numerous experimental studies have identified multiple mechanisms by which SNHL can lead to CI (Loughrey et al., 2018; Choi et al., 2021; Bikbov et al., 2022). In response to these findings, researchers have proposed three hypotheses on how SNHL brings about CI: the cognitive load (CL) hypothesis, the co-morbidity hypothesis, and the sensory deprivation hypothesis (Slade et al., 2020; Figure 1).

## CL hypothesis

The CL hypothesis suggests that the amount of information within the brain that can be processed, held in memory, and accessed at any given time is limited due to the limited availability of processing resources (Wingfield, 2016). ARHL results in patients receiving less information from the outside world. To achieve as much processing and preservation as possible of the limited amount of acoustic information, a higher demand is placed on the limited processing resources of patients with HL. This means that these patients need more cognitive resources for auditory perceptual processing. As a result, cognitive resources are diverted from other cognitive tasks to listening efforts, leading to a depletion of cognitive resources (Wingfield et al., 2005; Tun et al., 2009). This reallocation of resources has a detrimental effect on *CF* and could theoretically lead to a decrease in cognitive performance (Humes et al., 2013).

### SNHL and CL

Research to date regarding CL has found increases in neurovascular coupling responses as a result of CL (Csipo et al., 2021). One study (Luan et al., 2019) found that patients with SNHL showed a significant increase in distal functional coupling between the dorsolateral prefrontal cortex and the auditory cortex. Additionally, as the hearing status decreases, this coupling response becomes stronger. As is well established, the brain is naturally divided into four regions by the sulcal gyrus: the frontal, parietal, occipital and temporal lobes. The temporal lobe is currently thought to be primarily responsible for language function and auditory perception, as well as involved in long-term memory and emotion (Buchanan et al., 2006). The superior temporal gyrus, where the auditory center is located, is located in the part of the brain between the lateral and superior temporal sulci on the temporal lobe (Tae et al., 2014). In the brains of patients with HL, activities in and loads on the auditory center are increased to allow patients to better recognize acoustic signals, and correspondingly, other areas of cognitive reserve need to be called upon for auditory use, such as memory, emotion, language, and other areas (Hughes et al., 2018). Studies related to the use of hearing aids (Qian et al., 2016; Bucholc et al., 2022) and cochlear implants (Mertens et al., 2021) have demonstrated that as hearing aids and cochlear implants help to restore auditory perception, the onset of CI is delayed. It is hypothesized that hearing aids (Reinten et al., 2021) and cochlear implants (Chatterjee et al., 2023) improve the “effortful process” of sound discrimination in the daily lives of SNHL patients, and that this change reduces the previous over-allocation of cognitive resources, helping to restore balance in the CL.

### Neurotransmitters and CL

Research has demonstrated that the balance between excitation and inhibition in the brain is disrupted under different CLs (Bezalel et al., 2019). This balance is critical for the stability of cortical networks. Patients with ARHL have now been found to have reduced levels of gamma- aminobutyricacid (GABA) and glutamate (Glu) in the auditory center (Li et al., 2023). GABA is an important inhibitory neurotransmitter in the brain that is known to regulate inhibitory neurotransmission within the auditory system (Kotak et al., 2008). One study reported a correlation between the mean GABA level in the auditory cortex and mean binaural hearing thresholds, with greater HL associated with lower mean GABA levels. Further research (Dobri and Ross, 2021) found that older adults with ARHL have greater difficulty understanding speech in noisy environments as their GABA level declines. Bezalel et al. (2019) noted that GABA secretion in the dorsal anterior cingulate cortex (ACC), which plays a role in controlling behaviors (Vassena et al., 2020), is increased in high CL situations. GABA, however, is decreased in the auditory center of the temporal lobe in ARHL patients (Zemaitis et al., 2021), which does not contradict the previous decrease in total GABA. Interestingly, it has been suggested that increasing GABA in the auditory cortex or increasing the sensitivity of GABA receptors enhances the response of the auditory center to sound stimuli (Brecht et al., 2017). This reinforces the idea that HL causes a redistribution of CL, resulting in CI.

Additionally, some evidence is found in the literature that Glu also accumulates in the brains of animals with ARHL (Tadros et al., 2007; Kirschmann et al., 2019). Glu is the most abundant free amino acid in the brain as well as the main excitatory neurotransmitter in the brain. Glu can be released into the synaptic gap in a vesicular manner (Franco et al., 2021). Accumulation of Glu can increase CL in the auditory center and affect CL distribution. N-Methyl-D-aspartic acid receptors (NMDARs) are central mediators of glutamatergic neurotransmission and widely distributed, mainly regulating the inward flow of Ca^2+^ ions into neuronal cells and influencing neuronal activity (Joshi et al., 2019). This process is crucial in synaptic plasticity, which underlies activity-dependent learning and memory. Glu was found to be involved in regulating the channel opening of NMDARs (Wang et al., 2021), and overstimulation of NMDARs enhances the release of Glu via calcium channels. Under excitotoxic conditions, Glu leads to synaptic loss and elimination in hippocampal pyramidal cells (Companys-Alemany et al., 2022; Keimasi et al., 2023). It is now widely accepted that over-activation of NMDARs leads to the development of AD (Xu et al., 2021; Abad-Perez et al., 2023). However, recent studies have found that NMDARs are not only expressed in the cranial brain, but also in inner hair cells (IHCs), and that overactivation of NMDARs also results in Glu release in IHCs (Tang et al., 2014; Kaur et al., 2020; Song et al., 2021). Excessive Glu release causes excitatory neurotoxic effects, reducing the number of ribbon synapses in the cochlea and altering synaptic morphology, resulting in impaired signaling of the cochlear nerve and affecting the patient’s hearing (Hong et al., 2018). NMDARs are involved in the regulation of neurogenesis and spatial memory formation, with the NR2A and NR2B subunits playing crucial roles (Hu et al., 2008; Sun et al., 2011). Bone morphogenic protein 4 (BMP4) is a member of the transforming growth factor-β (TGF-β) family, which is involved in the regulation of cell proliferation and survival. Chen et al. (2014) found that BMP4 regulates cochlear epithelial cell survival by altering the expression of the NR2B subunit of NMDARs. Song et al. (2021) and Ralli et al. (2014) found that administration of NMDA channel blockers in their experimental animal model delayed the onset of salicylic acid-induced DRHL and tinnitus. α-amino-3-hydroxy-5-methyl-4-isoxazole propionic acid receptors (AMDARs) are Glu-gated ion channels that mediate most of the rapid excitatory synaptic transmission in the brain (Diering and Huganir, 2018). In addition to NMDARs, studies have shown that AMDARs are also modulated by neurotransmitters (Brechet et al., 2017). Previous research has revealed that CI results in a reduction of synaptic AMPAR in the hippocampus (Chang et al., 2006). Specifically, in the cochlea, AMDAR is primarily located in the nerve endings near the base of the IHCs (Hong et al., 2018), Furthermore, in the cochlea, AMDAR plays a crucial role in mediating rapid excitatory transmission at mature spiral ganglion neurons SGNs afferent synapses (Lozier et al., 2023). Interestingly, changes in the subunit composition of synaptic AMDAR occur as a result of HL, leading to long-term effects on synaptic integration (Pilati et al., 2016).

## Co-morbidity hypothesis

The co-morbidity hypothesis, or common etiology hypothesis, presumes that CI and SNHL are due to a common cause. Clinically, physicians have found by magnetic resonance imaging (MRI) that the volume of temporal gray matter (TGM) is significantly less in patients with AD than in normal older adults (Li et al., 2020), and the same shrinkage of TGM occurs in patients with ARHL (Armstrong et al., 2019; Slade et al., 2022).

### Pathological manifestations

The limbic system (LS; Kalus et al., 2006) includes the pear-shaped cortex, internal olfactory area, orbital gyrus, cingulate gyrus (CG), subcallosal gyrus, hippocampal gyrus, insula, temporal pole, amygdala, septum, preoptic area, hypothalamus, hippocampus, papillae, etc. The LS is extensively interconnected with the rest of the nervous system and is closely associated with sensation, regulation of visceral activity, emotion, behavior, learning, and memory (Manchella et al., 2023). The CG consists mainly of the ACC and posterior cingulate cortex (PCC). Adults with ARHL show significant volume atrophy in the ACC, PCC, precentral gyrus, postcentral gyrus, and parahippocampus on MRI in comparison to normal individuals (Belkhiria et al., 2019). The PCC plays an important role in CFs such as episodic memory, spatial attention, and self-evaluation. The precentral gyrus, located in the frontal lobe, is the highest somatic motor center and is responsible for movement of the contralateral limbs. The postcentral gyrus is located in the parietal lobe and is the highest sensory center. The parahippocampus is also part of the LS, and previous studies have shown that they are closely related to emotion regulation (Kleen et al., 2010). In addition to the reduced volume of the TMG and LS, Paciello et al. (2023) found that the auditory cortex (ACx) is damaged in a mouse model of ARHL, and Xu et al. (2023) found that rats given noise stimulation for 6 months exhibited not only binaural HL but also damage to the ACx.

Amyloid plaques formed by β-amyloid (Aβ) and neurofibrillary tangles formed by abnormally modified tau proteins are the hallmarks of AD (Skouras et al., 2020; Ossenkoppele et al., 2022; Tautou et al., 2023). Aβ is a peptide produced by hydrolysis of amyloid precursor protein (APP), and excess Aβ accumulation in mitochondria activates astrocytes and microglia, damages neurons (Huang et al., 2023), and induces mitochondrial autophagy, promoting reactive oxygen species (ROS) production and accelerating neural oxidation (Dou and Tan, 2023). At the same time, over-phosphorylation of tau protein eliminates its ability to form and maintain stable microtubules, reduces the dissociation of microtubule protein molecules, and induces microtubule bundling (Kandimalla et al., 2018; Solas et al., 2023; Zhang et al., 2023). In turn, this affects neuronal cell signaling and the mitosis of other cell types in the brain. Notably, some studies have found that Aβ and tau protein levels are significantly elevated in the brain of HL patients (Xu et al., 2019; Golub et al., 2021; Irace et al., 2022; Wang et al., 2022; Zheng et al., 2022). Consistently, both Aβ and tau were found to be significantly elevated in patients with AD plus HL (Zhang et al., 2022).

### Potential mechanisms

Although current clinical studies have found a link between SNHL and CI, no clear pathway for this process has been established. The co-morbidity hypothesis suggests that pathways such as oxidative damage, neuroinflammation and accumulation of harmful substances may be a common cause of both SNHL and CI.

#### Oxidative damage and neuroinflammation

Oxidative damage (Maurya et al., 2022) and neuroinflammation have long been recognized as significant features of neurodegenerative diseases. Previous research has indicated that oxidative damage can impact the activity and expression of DNA methyltransferases. Specifically, an elevated expression of DNA methyltransferase 1 (DNMT1) has been linked to memory impairment in amnesic mice (Srivas and Thakur, 2017). Case reports have also documented that mutations in DNMT1 can lead to hereditary sensory and autonomic neuropathy with CI (Klein et al., 2011).

Adenosine is an endogenous purine nucleoside, and adenosine receptors, including A1R, A2R and A3R, are involved in the regulation of neurotransmitter release, oxidative stress responses, inflammation, blood flow, and a variety of intracellular signaling pathways including apoptosis. Activation of A1R protects inner ear hair cells, reduces hair cell death, and effectively protects against noise damage (Kaur et al., 2016; Chang et al., 2017; Fok et al., 2020). Inhibition of A1R specifically was shown to cause cochlear nerve damage and to increase susceptibility to HL (Vlajkovic et al., 2017). However, intracranially, A1R has a neuroprotective effect (Shi et al., 2021), and disruption of A1R exacerbates long-term potentiation of the hippocampus, which can cause CI (Zhang et al., 2020). A2R represents a class of adenosine receptors that have been extensively studied and found to be associated with pathophysiological conditions such as inflammatory diseases and neurodegenerative disorders. In contrast to the neuroprotective effect of A1R in the ear, inhibition of adenosine A2A receptor (A2AR) increases the resistance of the cochlea to acoustic damage (Vlajkovic et al., 2017; Shin et al., 2021; Oliveros et al., 2022). Within the cranium, activation of A2AR promotes neuroinflammation, reducing synaptic plasticity (Merighi et al., 2022; Chen et al., 2023). However, adenosine A2B receptor was shown to have the opposite effect of A1R (Qiang et al., 2021).

Pattern recognition receptors (PRRs) are important components of the body’s innate immune system, are widely distributed, and are present in a variety of forms. In the brain, glial cells are known to express a variety of PRRs. Toll-like receptors (TLRs) are one type of PRR. Different TLRs determine not only which ligands are recognized but also the nature of the signals generated (Blasius and Beutler, 2010). Among the many TLRs, TLR4 in particular has been studied more in HL (Müller, 2020). TLR4 is activated primarily by lipopolysaccharide from Gram-negative bacteria (Bowyer et al., 2020) and sequentially triggers the immune response of the body, and studies have found that overexpression of TLR4 in the brain activates the nuclear factor kappa B (NF-κB) signaling pathway (Wang et al., 2021; Abd El-Rahman and Fayed, 2022; Kwon and Lee, 2022), exacerbating the inflammatory response, activating microglia, damaging neurons, and causing CI (van Well et al., 2012; Zhang et al., 2018; Campolo et al., 2019; Potter et al., 2019; Tang et al., 2021; Islam et al., 2022). However, other studies have provided evidence that mild activation of TLR4 can enhance the phagocytic capacity of glial cells, clear accumulated Aβ early, and delay the onset of CI (Qin et al., 2016; Wu et al., 2022). In addition to its expression in brain tissue, TLR4 is also expressed in cochlear tissue (Zhang et al., 2019) and, upon lipopolysaccharide stimulation, activates a range of immune responses via the NF-κB signaling pathway (Si et al., 2015; Liu et al., 2020). A recent study found that overexpression of TLR4 causes a significant increase in HL (Zhang et al., 2019). Therefore, we suggest that the process of chronic immune activity induced by the TLR4-mediated NF-κB signaling pathway may be one of the potential mechanisms responsible for HL with CL.

#### Accumulation of harmful substances

The interstitial fluid and cerebrospinal fluid are two extracellular fluids present in the cranium. These two extracellular fluids not only provide protective buffering for the brain, but also are involved in the transport of nutrients and waste products, the maintenance of electrolyte homeostasis (Voisin et al., 1999), and signal transduction. Maintenance of this homeostasis in the brain is regulated by the aquaporins (AQPs; Benga and Huber, 2012). The AQPs are cell membrane proteins with the main function of controlling the movement of water in and out of the cell (Voisin et al., 1999). Aquaporin 4 (AQP4) is the major AQP found in the brain where it plays a significant role in water homeostasis. Current research has established that diminished AQP4 expression leads to reduced Aβ clearance and causes Aβ accumulation, leading to deficiencies in memory and learning ability and the development of CI (Hubbard et al., 2018; Chandra et al., 2021; Fang et al., 2021; Liu et al., 2022; Wang et al., 2022; Vasciaveo et al., 2023). In addition to regulating water homeostasis, AQP4 also activates astrocytes (Yang et al., 2022; Liu et al., 2023), and outside of the brain, it is expressed by IHCs (Christensen et al., 2009; Nishio et al., 2013). AQP4 is now widely believed to be involved in maintaining the osmotic balance during the K^+^ cycle as well as the ionic balance of the endolymphatic fluid (Li and Verkman, 2001; Mhatre et al., 2002). AQP3 and AQP5 are also expressed in the cochlea and, like AQP4, are involved in regulating the ionic balance of endolymphatic fluid. Research studies have proposed that AQPs are closely associated with Ménière’s disease (Eckhard et al., 2014, 2015; Nevoux et al., 2015).

In addition to TLRs, the nucleotide-binding oligomerization domain-like receptor family is the most representative class of PRRs. NLR pyrin domain-containing 3 (NLRP3) is a member of this family that is associated with many diseases. Studies have shown that microglia-mediated activation of NLRP3 is closely related to cognition (Guo et al., 2020; Feng et al., 2021). Mitochondrial damage or production of mitochondrial ROS (mtROS) is an important regulator of NLRP3 activation. mtROS activates NLRP3 in microglia, which in turn promotes activation of caspase-1, a downstream protein of NLRP3, and the secretion of IL-1β and IL-18, which in turn stimulates a neuroinflammatory response (Tian et al., 2021; Barczuk et al., 2022; Liang et al., 2022; Li et al., 2023; Su et al., 2023). Two trials have reported that treatment with NLRP3 inhibitors is effective at alleviating the onset of CI (Zheng et al., 2022; Lin et al., 2023). NLRP3 has also been found to be associated with both AD and HL. In both genetic deafness (Nakanishi et al., 2018; Ma et al., 2022) or SNHL (Kim et al., 2021), NLRP3 is thought to induce inflammatory damage in IHCs and SGNs via ROS activation and to reduce the occurrence of autophagy. The study by Sai et al. (2022) showed that noise exposure activates NLRP3 inflammation in the cochlea and increases the production of IL-18 and IL-1β, inducing inflammation in the cochlea. Tang et al. (2021) found that exogenous application of the chemical BDE-47 activates ROS and NLRP3 inflammatory vesicles in cochlear hair cells as well as the p38 MAPK pathway, causing HL.

## Sensory deprivation hypothesis

The sensory deprivation hypothesis shares some conceptual commonalities with the CL hypothesis, but it places greater emphasis on the long-term reallocation of cognitive resources toward hearing in patients with SNHL due to chronic sensory deprivation, which leads to cognitive decline (Lindenberger and Baltes, 1994; Humes et al., 2013). This hypothesis highlights that prolonged sensory deprivation leads to compensatory cortical reorganization and neural alterations that hinder general cognitive and affective processes. Previous studies have provided evidence supporting cortical alterations in ARHL, including an increased reliance on frontal brain regions during speech perception (Du et al., 2016; Rosemann and Thiel, 2018) and a reduction in gray matter in the auditory cortex caused by diminished hearing ability (Eckert et al., 2019). While inadequate sensory input directly affects cognition through deprivation, it may also indirectly impact cognition through reduced socialization and communication or increased depression (Dawes et al., 2015; Stahl, 2017). This hypothesis suggests that decreased social interaction associated with social isolation and depression may mediate the causal relationship between HL and cognitive decline (Dawes et al., 2015; Whitson et al., 2018). Indeed, significant associations have been found between depressive symptoms, heightened social isolation, and diminished quality of life among patients with SNHL (Panza et al., 2019). According to this perspective, neural changes resulting from SNHL, such as reduced ACC activation, can directly influence mood and emotion regulation (Husain et al., 2014). The anterior ventral location of the ACC within the corpus callosum plays a crucial role in depressive symptoms, and thus, a reduction in ACC volume can lead to impaired emotion processing (Belkhiria et al., 2019).

### HL inflect mental illness

A significant relationship was identified between HL and emotional loneliness (Jayakody et al., 2022). A study in the United Kingdom reported that the adverse effects of HL are not limited to hearing impairment but may also include negative effects on psychosocial health (Tsimpida et al., 2022). In another study, middle-aged and elderly patients with HL were more likely to have diminished health status, depression, and suicidal ideation compared with participants without HL (Park et al., 2022). Additional studies (Chern et al., 2022; Huang et al., 2022) have suggested that HL can contribute to psychosocial disorders in patients. An MRI study revealed that gray matter volume in the middle cingulate cortex is positively correlated with high-frequency hearing impairment in patients with ARHL (Ma et al., 2022). These results suggest that HL can influence mental health.

## Conclusion

An epidemiological investigation estimated that by 2023, 6.7 million Americans aged 65 years and older would have AD and that 73% of Americans 75 years or older would be affected (Alzheimer's and dementia: the journal of the Alzheimer's Association, 2023). In this review, we explore three hypotheses for the co-occurrence of SNHL and CI: the CL hypothesis, the co-morbidity hypothesis, and the sensory deprivation hypothesis. The CL hypothesis emphasizes that when acoustic signals are received, the CL is redistributed in the brain of patients with SNHL, increasing the burden on the auditory center and resulting in a constant high load on the auditory center, leading to a decrease in CL elsewhere and a decline in *CF.* The co-morbidity hypothesis suggests that SNHL and CI are diseases of the same type and occur due to a common cause. Indeed, SNHL and CI are extremely similar in terms of pathological changes, including volumetric atrophy of functional brain areas and deposition of the toxic and harmful substances Aβ and tau. Chronic neuroinflammation and long-term oxidative damage are now considered to be the common cause of their pathogenesis. In addition, accumulation of toxic substances and alterations in ion channel expression also may play a role in the development of these conditions. Both the general CL hypothesis and the co-morbidity hypothesis address the direct causes of SNHL and CI. In contrast, the sensory deprivation hypothesis proposes that an indirect pathway contributes to CI in patients with SNHL. The sensory deprivation hypothesis suggests that long-term auditory decline affects people’s psycho-spiritual health, increases their sense of isolation, and increases their risk of psycho-spiritual disorders. However, it is currently believed that psychosocial illness and CI are mutually reinforcing. Long-term auditory decline contributes to a high risk of psychosocial illness and increases the risk of CI. We propose that the co-occurrence of SNHL and CI is the result of a combination of direct and indirect causes.

However, there are limitations to the three hypotheses mentioned above. While there is some evidences for all three hypotheses, these evidences are fragmented and still lack a relatively complete basis. The sensory deprivation hypothesis suggests that the absence of sensation causes cortical changes, however, one study found a limited effect of HL on these changes (Parker et al., 2020). CL is thought to be exacerbated by hearing impairment, but this change has also been noted in some cases of visual impairment (Golzan et al., 2017; Vu et al., 2021). Age has long been recognized as one of the main causative factors in the co-morbidity hypothesis; however, aging can lead to a variety of diseases and sensory loss, such as cardiovascular disease (Yang et al., 2023), cerebrovascular disease (Romay et al., 2024), and vision decline (Vanhunsel et al., 2021). All of these diseases can lead to CI and these findings seem to support that age, rather than ARHL, is the underlying cause of CI.

A survey of populations in the UK and France found that HL is significantly and positively associated with an increased risk of AD during an exposure window of 2–10 years prior to AD diagnosis (Nedelec et al., 2022). Despite the limitations of the three hypotheses mentioned in this review, they are still the prevailing viewpoints and have implications for the study of SNHL and CI. As the incidence of SNHL increases each year, we should recognize the dangers of this disabling disease. Hearing aids and cochlear implants have been shown to be effective at improving hearing and delaying the onset of CI. As these devices improve a patient’s hearing, they not only reduce the load on the auditory center but also improve the patient’s ability for interpersonal communication, which can reduce their sense of isolation and risk of psychosocial disorders.

## Author contributions

HZ: Formal analysis, Resources, Validation, Writing – original draft. YW: Data curation, Formal analysis, Resources, Writing – original draft. LCu: Formal analysis, Funding acquisition, Investigation, Resources, Writing – review & editing. HW: Data curation, Formal analysis, Resources, Writing – review & editing. SL: Data curation, Resources, Validation, Writing – review & editing. TL: Resources, Supervision, Writing – review & editing. DL: Data curation, Supervision, Writing – review & editing. LCh: Data curation, Funding acquisition, Resources, Validation, Writing – review & editing. JQ: Conceptualization, Formal analysis, Funding acquisition, Validation, Writing – review & editing. YS: Conceptualization, Funding acquisition, Resources, Supervision, Validation, Writing – review & editing.
